# The *Candida* species that are important for the development of atrophic glossitis in xerostomia patients

**DOI:** 10.1186/s12903-017-0449-3

**Published:** 2017-12-16

**Authors:** Sachika Nakamura, Mariko R. Okamoto, Ken Yamamoto, Akihisa Tsurumoto, Yoko Yoshino, Hiroshi Iwabuchi, Ichiro Saito, Nobuko Maeda, Yoichi Nakagawa

**Affiliations:** 1 Department of Clinical Pathophysiology., Tsurumi University School of Dental Medicine, Tsurumi University Dental Hospital, 2-1-3 Tsurumi, Tsurumi-ku, Yokohama, 230-8501 Japan; 2 Department of Oral Microbiology, Tsurumi University, School of Dental Medicine, Yokohama, Japan; 3 Kobayashi Dental Clinic, Niigata, Japan; 4 Community Dentistry, Tsurumi University School of Dental Medicine, Yokohama, Japan; 5 Department of Nutrition and Dietetics, Sagami Women’s University, Sagamihara, Japan; 6 Department of Dentomaxillofacial Diagnosis and Treatment, Division of Oral and Maxillofacial Surgery, Graduate School of Kanagawa Dental University, Yokosuka, Japan; 7 Department of Pathology, Tsurumi University School of Dental Medicine, Yokohama, Japan

**Keywords:** Atrophic glossitis, *Candida* species, Candidiasis, Xerostomia, Logistic regression analysis

## Abstract

**Background:**

The purpose of this study was to clarify the species of *Candida* that are important for the development of atrophic glossitis in xerostomia patients.

**Methods:**

A total of 231 patients with subjective dry mouth were enrolled in the present study. Logistic regression analysis was performed to clarify the contribution of each *Candida* species and other variables to the development of atrophic glossitis. The dependent variable was the absence/presence of atrophic glossitis. The *Candida* colony-forming units (CFU) of *C. albicans*, *C. glabrata*, *C. tropicalis*, and *C. krusei*, as well as age, gender, resting (RSFR) and stimulated (SSFR) whole salivary flow rate, and denture-wearing status, were treated as explanatory variables.

**Results:**

Logistic regression analysis showed that two factors were closely associated with the presence of atrophic glossitis: an increase in *C. albicans* CFU and a decrease in the SSFR.

**Conclusions:**

*C. albicans*, but not non-*albicans Candida*, was associated with atrophic glossitis in xerostomia patients who had no systemic predisposing factors, indicating that *C. albicans* remains a treatment target for *Candida*-related atrophic glossitis.

## Background

Oral candidiasis is sub-classified into three major variants: pseudomembranous, erythematous, and hypertrophic [1, 2]. In addition to these variants, angular cheilitis, median rhomboid glossitis, and denture stomatitis have been recognized as *Candida*-associated lesions; moreover, *Candida* super-infection in oral lichen planus is frequently encountered. The various forms of oral candidiasis are caused by interactions between the host defenses and fungal virulence factors.

Atrophic glossitis is caused by complete or partial lingual papillary atrophy. It exhibits a smooth, glossy appearance with a red or pink background and is primarily a manifestation of an underlying condition. Nutritional deficiencies (iron, folic acid, vitamin B_12_, riboflavin, and niacin) are the most common causes [3]. Other etiological factors of atrophic glossitis include hyposalivation and candidiasis [2, 4–6]. An increase in *Candida* colony counts, a low salivary flow rate, and advanced age were identified as being risk factors that were closely associated with the development of atrophic glossitis [7, 8]. However, the *Candida* species that are the most relevant to the development of atrophic glossitis remain unclear.

*Candida albicans* is the primary causative organism of oral candidiasis. Recent studies have suggested that non-*albicans Candida* (NAC) species, such as *C. glabrata*, *C. parapsilosis*, *C. tropicalis*, and *C. krusei* are also pathogenic in humans [2, 8–13]. One of these studies showed that *C. albicans* was frequently isolated from the tongue, while *C. glabrata* was most frequently isolated from the angle of the mouth, suggesting that certain *Candida* species are associated with oral candidiasis at particular sites [8]. In a study on the relationship between *Candida* species and local and systemic predisposing factors, only *C. albicans* infections were significantly associated with the use of inhaled steroids and antibiotics and super-infection with oral lichen planus, whereas the presence of removable dentures was significantly associated with the detection of NAC or a mixed flora consisting of *C. albicans* and NAC [9]. Similarly, the incidence of colonization with more than one *Candida* species was found to be higher in cases of denture stomatitis than in cases involving other forms of oral candidiasis [10]. *C. albicans* and *C. glabrata* were the most prevalent species detected in cases of mixed infections [10, 14]. However, no association has been confirmed between a specific *Candida* species and any particular sign or symptom of oral candidiasis.

The purpose of this study was to clarify the species of *Candida* that are important for the development of atrophic glossitis in xerostomia patients.

## Methods

### Patient selection

The subjects enrolled in the study were patients at the Dry Mouth Clinic at Tsurumi University Dental Hospital. Patients who had subjective dry mouth (xerostomia) were examined to determine their salivary flow rate and oral findings, and a mycological examination was performed. The examinations were performed between January 2010 and February 2013. Patients with systemic predisposing factors for candidiasis, such as diabetes mellitus, chronic obstructive pulmonary disease, immunodeficiency, and malnutrition, and patients who were using medications that predisposed them to candidiasis, such as antibiotics and steroids, were excluded from this study. A total of 231 patients (mean age ± standard deviation, 66.6 ± 12.6 years; range 33 to 96 years), were enrolled in this study. Forty-two of the patients were men (18.2%), and 189 were women (81.8%).

### Evaluation of atrophic glossitis

**Fig. 1:**
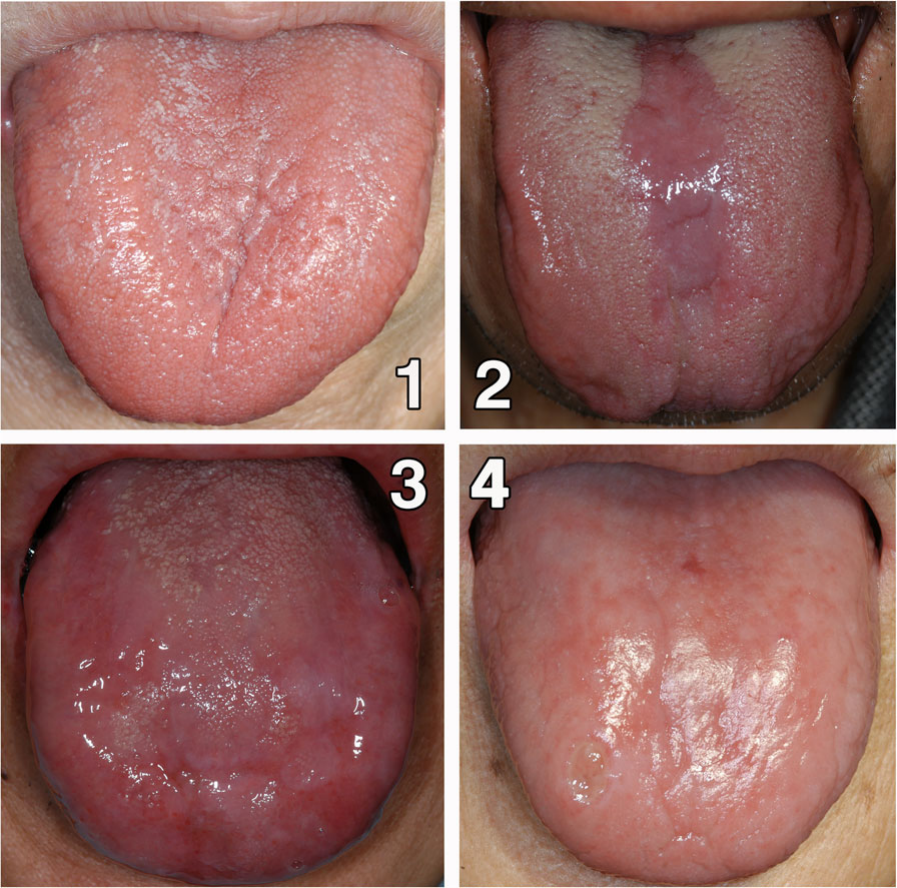
The grades of atrophic glossitis. 0, none; 1 (mild), the lingual papillae in the affected area of the tongue were smaller in size than those in the intact area; 2 (moderate), partial loss (<50%) of the lingual papillae on the dorsum of the tongue; 3 (severe), partial loss (>50%) of the lingual papillae on the dorsum of the tongue; and 4 (profound loss)

The presence of atrophic glossitis was determined by the attending dentist. The severity and extent of papillary atrophy and loss were graded from 0 to 4 as follows: 0, none; 1, mild; 2, moderate; 3, severe; and 4, profound loss (Fig. 1). Atrophic glossitis was considered mild when the lingual papillae in the affected area of the tongue were smaller in size than in the intact area. Partial loss (<50%) of the lingual papillae of the dorsum of the tongue was considered to indicate moderate disease, and partial loss (>50%) was considered to indicate severe disease. Patients who showed loss of the lingual papillae throughout the dorsum of the tongue were considered to have profound loss. When the attending dentist could not determine the amount of papillary atrophy, a consensus was reached by discussion with an oral surgeon (Y.N.) with 30 years of experience [7].

### Salivary secretion test

To measure the resting whole salivary flow rate (RSFR), each subject sat at rest, and saliva was collected into a cup during a 15-min period in the absence of masticatory movements. The saliva volume was measured using a disposable syringe. An RSFR of ≤1.5 mL/15 min (≤ 0.1 mL/min) was classified as hyposalivation according to previously determined criteria [15, 16]. The stimulated whole salivary flow rate (SSFR) was also measured by collecting saliva during a 10-min period. Salivation was stimulated by chewing a piece of gum (Free zone gum; Lotte Co., Ltd., Tokyo, Japan). An SSFR of ≤10 mL/10 min was classified as hyposalivation.

### Fungal culture

The dorsum of the tongue was swabbed 10 times with a cotton applicator (Eiken Chemical Co., Ltd., Tokyo, Japan), and the swab was directly inoculated onto CHROMagar™ Candida selective plates (CHROMagar Microbiology, Paris, France) [7, 17]. The number of *Candida* colonies was counted after incubation at 30 °C for 48 h and was expressed as colony-forming units (CFU) per plate. Each *Candida* species was identified by colony color in accordance with the manufacturer’s manual [17].

### Statistical analyses

A multivariate logistic regression analysis was used to clarify the contribution of each *Candida* species and the other variables to the development of atrophic glossitis. The dependent variable was the absence (Grade 0)/presence (Grades 1–4) of atrophic glossitis, while the *Candida* CFU of *C. albicans*, *C. glabrata*, *C. tropicalis*, and *C. krusei* as well as age, RSRF, SSRF and denture-wearing status were treated as explanatory variables. The results of the logistic regression analysis were used to ascertain which independent variables had a major effect on the absence or presence of atrophic glossitis.

Salivation and the patient’s denture-wearing status were analyzed as factors associated with increased *Candida* colony counts. To determine the effects of hyposalivation and denture wearing on *Candida* colony counts, differences among the groups were evaluated by the Kruskal-Wallis H test, followed by multiple comparison testing using the Mann-Whitney U test with Bonferroni correction. A total of four groups were formed through combinations of two factors: salivation status (hyposalivation [SSFR ≤10 mL/10 min] or normosalivation [SSFR >10 mL/10 min]) and denture-wearing status (denture wearing/no dentures).

All statistical analyses were performed using the SPSS software program, ver. 22.0 J (IBM Japan, Tokyo). *P* values of <0.05 were considered to indicate statistical significance.

## Results

### Study population

Overall, atrophic glossitis was noted in 46 patients (Table 1), tongue redness in 58, palate redness in 16, buccal mucosa redness in 10, lip redness in 16 and angular cheilitis in 25. The mean RSFR was 2.1 ± 2.7 mL/15 min (range, 0–16.0 mL/15 min); 26 (11.3%) patients had an RSFR of 0 mL/15 min, and 141 (61.0%) patients had an RSFR of ≤1.5 mL/15 min. The mean SSFR was 11.3 ± 7.0 mL/10 min (range, 0.6–40.6 mL/10 min); 109 (47.2%) patients had an SSFR ≤10 mL/10 min. According to the Japanese criteria [18], 26 (11.3%) patients were diagnosed with Sjögren’s syndrome. Ninety-two (39.8%) patients wore dentures. Partial removable dentures were more commonly used than full dentures, but we did not consider the type of denture in our analysis.

**Table 1:** Characteristics of study population

Atrophic glossitis	Number	Age	Male:Female	Saliva flow rate		Denture wearer
				RSFR	SSFR	
Grade 0	185	63.4 ± 12.5	37:148	2.4 ± 2.7	12.2 ± 6.9	67/185 (36.2%)
Grade 1	15	72.8 ± 10.8	1:14	1.9 ± 3.3	11.3 ± 6.1	8/15 (53.3%)
Grade 2	23	68.2 ± 11.9	2:21	1.0 ± 2.4	6.3 ± 6.2	11/23 (47.8%)
Grade 3	5	74.4 ± 10.0	2:3	1.1 ± 0.7	7.7 ± 4.8	4/5 (80.0%)
Grade 4	3	83.0 ± 6.5	0:3	0.1 ± 0.1	3.9 ± 0.8	2/3 (66.7%)
Total	231	66.6 ± 12.6	42:189	2.1 ± 2.7	11.3 ± 7.0	92/231(39.8%)

*RSFR* resting whole salivary flow rate (mL/15 min), *SSFR* stimulated whole salivary flow rate (mL/10 min); Values are mean ± SD

### *Candida* colonization according to the severity of atrophic glossitis

One hundred thirty-five (58.4%) patients were *Candida*-positive (Table 2). The species of *Candida* in these cases were as follows: *C. albicans, n* = 119 (88.1%); *C. glabrata, n* = 50 (37.0%); *C. tropicalis, n* = 20 (14.8%), and *C. krusei*, *n* = 9 (6.7%). *C. albicans* and *C. glabrata* were simultaneously detected in 41 (30.4%) of the 135 patients.

**Table 2:** Prevalence of *Candida* colonization according to the severity of atrophic glossitis

Atrophic glossitis	Detection of *Candida* ^a^				
	*C. albicans*	*C. glabrata*	*C. tropicalis*	*C. krusei*	*Candida* spp.
Grade 0	76/185	30/185	16/185	5/185	14/185
Grade 1	13/15	4/15	3/15	0/15	0/15
Grade 2	22/23	11/23	1/23	4/23	3/23
Grade 3	5/5	2/5	0/5	0/5	0/5
Grade 4	3/3	3/3	0/3	0/3	1/3
Total	119/231	^b^50/231	20/231	9/231	18/231

^a^The culture test was positive in 135/231 (58.4%) patients. Values are number of cases

^b^ *C. albicans* and *C. glabrata* were simultaneously detected in 41 cases

### The variables of patients with and without atrophic glossitis

When the participants were divided into two groups according to the absence/presence of atrophic glossitis (Table 3), the number of *C. albicans* CFU in the group with atrophic glossitis was found to be significantly higher (255.6 ± 750.0 CFU) than that in the group without atrophic glossitis (35.2 ± 92.3 CFU) (*P* < 0.001). The *C. glabrata* CFU in the group with atrophic glossitis (37.9 ± 115.2 CFU) was significantly lower than that in the group without atrophic glossitis (45.8 ± 278.7 CFU) (*P* < 0.001).

**Table 3:** Comparison of variables between absent (Grade 0) and present (Grade1~4) groups of atrophic glossitis

	Atrophic glossitis		*P*-value
	Grade 0	Grade 1~4	
	*n* = 185	*n* = 46	
*C. albicans* (CFU)	35.2 ± 92.3	255.6 ± 750.0	<0.001
*C. glabrata* (CFU)	45.8 ± 278.7	37.9 ± 115.2	<0.001
*C. tropicalis* (CFU)	0.6 ± 5.8	3.9 ± 18.6	0.058
*C. krusei* (CFU)	0.9 ± 6.3	0.3 ± 1.2	0.974
RSFR (mL/15 min)	2.4 ± 2.7	1.3 ± 2.6	<0.001
SSFR (mL/10 min)	12.2 ± 6.9	7.9 ± 6.4	<0.001
Age	65.4 ± 12.5	71.4 ± 12.0	0.004

Values are expressed as Mean ± SD. *P*-value, Mann-Whitney U-test

*RSFR* resting whole salivary flow rate, *SSFR* stimulated whole salivary flow rate

In the group with atrophic glossitis, the mean RSFR was 1.3 ± 2.6 mL/15 min, while the mean SSFR was 7.9 ± 6.4 mL/10 min. In contrast, in the group without atrophic glossitis, the mean RSFR was 2.4 ± 2.7 mL/15 min, and the mean SSFR was 12.2 ± 6.9 mL/10 min. Thus, both the mean resting (*P* < 0.001) and stimulated (*P* < 0.001) saliva flow rates were significantly lower in the patients with atrophic glossitis.

A chi-squared test revealed that the gender of the groups with and without atrophic glossitis did not differ to a statistically significant extent (*P* = 0.211; Table 4). The chi-squared test also revealed that denture wearing was associated with atrophic glossitis (*P* = 0.025).

**Table 4:** Comparison of gender and denture-wearing status between absent (Grade 0) and present (Grade1~4) groups of atrophic glossitis

		Atrophic glossitis		Total	*P*-value
		Grade 0	Grade 1~4		
Gender	Male	37	5	42	0.221
	Female	148	41	189	
	Total	185	46	231	
Denture	Wearing	67	25	92	0.025
	No dentures	118	21	139	
	Total	185	46	231	

Values are number of cases. *P*-value, Chi square test

*RSFR* resting whole salivary flow rate, *SSFR* stimulated whole salivary flow rate

### Correlations among the colonizing species of *Candida*, age, and salivary flow rate

Significant relationships were observed between age and the amounts of *Candida* species as well as between age and the RSFR according to the Spearman’s rank correlation coefficients (Table 5). Significant relationships were also observed among the *Candida* species, except between *C. albicans* and *C. krusei*, which showed weak relationships. Negative correlations were observed between the amounts of *Candida* species and the salivary flow rate. There was no significant correlation between *C. krusei* and the salivary flow rate.

**Table 5:** Correlations among amount of *Candida* species, age, and salivary flow rate

	*C. albicans*	*C. glabrata*	*C. tropicalis*	*C. krusei*	RSFR	SSFR
Age	0.296**	0.288**	0.175**	0.155*	−0.210**	−0.099
*C. albicans*		0.345**	0.157*	0.039	−0.430**	−0.351**
*C. glabrata*			0.189**	0.246**	−0.320**	−0.269**
*C. tropicalis*				0.178**	−0.248**	−0.278**
*C. krusei*					−0.079	−0.031
RSFR						0.701**
SSFR						

Values represent Spearman’s rank correlation coefficients; *P < 0.05, **P < 0.001

*RSFR* resting whole salivary flow rate, *SSFR* stimulated whole salivary flow rate

### Factors associated with atrophic glossitis

Logistic regression analysis determined the factors that were closely associated with the presence of atrophic glossitis (Table 6). These factors included an increase in *C. albicans* CFU (Odds ratio, 1.004; *95% CI*, 1.001–1.007) and a decrease in SSFR (Odds ratio, 0.919; *95% CI*, 0.845–0.999); *C. albicans* showed the greatest contribution to atrophic tongue. Although *C. glabrata* was detected in 50 of 135 (37.0%) patients and was the second-most common species after *C. albicans* (Table 2), *C. glabrata* was not significantly associated with the presence of atrophic glossitis (Table 6).

**Table 6:** Predictors of atrophic glossitis -Multivariate logistic regression analysis-

	Coefficient	*P*-value	Odds ratio	*95% CI*	
				Lower	Upper
Age	0.029	0.135	1.029	0.991	1.069
Gender	0.804	0.196	2.234	0.661	7.549
*C. albicans* (CFU)	0.004	0.004	1.004	1.001	1.007
*C. glabrata* (CFU)	−0.001	0.366	0.999	0.996	1.001
*C. tropicalis* (CFU)	0.015	0.353	1.015	0.983	1.048
*C. krusei* (CFU)	−0.055	0.581	0.946	0.778	1.151
RSFR (mL/15 min)	0.045	0.675	1.047	0.846	1.295
SSFR (mL/10 min)	−0.085	0.048	0.919	0.845	0.999
Denture	0.364	0.389	1.439	0.629	3.294
Constant	−3.752	0.010	0.023		

*RSFR* resting whole salivary flow rate, *SSFR* stimulated whole salivary flow rate

Gender; female: 1, male: 0, Denture; denture wearing: 1, no dentures: 0

### The influences of the stimulated salivary flow rate and denture wearing on the amounts of *Candida* colonies

**Fig. 2:**
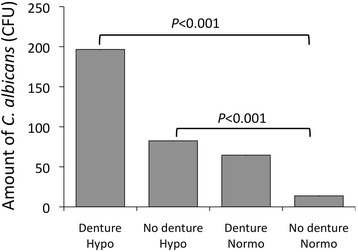
The associations between salivation and denture-wearing status and *C. albicans* colonization. The patients were divided into four groups according to their salivation status (normosalivation [Normo; SSFR >10 mL/10 min] or hyposalivation [Hypo; SSFR ≤10 mL/10 min]) and denture-wearing status (denture wearing or no dentures). The bar graph shows the *C. albicans* colony-forming units (CFU) stratified by salivation and denture-wearing statuses. The columns show the means. The Kruskal-Wallis H test, followed by multiple comparison testing using the Mann-Whitney U test with Bonferroni correction, was used to test statistical significance

Logistic regression analysis demonstrated that denture wearing was not associated with atrophic glossitis (Table 6). The question then arose as to whether denture wearing promoted an increase in the number of *C. albicans* CFU. We therefore examined the relationships between hyposalivation and denture wearing and the amount of *C. albicans* using the Kruskal-Wallis H test, followed by multiple comparison testing using the Mann-Whitney U test with Bonferroni correction (Fig. 2). Both denture-wearing (mean CFU ± standard deviation, 199.6 ± 737.0 CFU) and non-denture-wearing hyposalivation patients (82.5 ± 173.1 CFU) showed a significantly high number of *C. albicans* colonies compared with the non-denture-wearing normosalivation patients (13.8 ± 42.4 CFU). There was no significant difference in the number of *C. albicans* colonies between the denture-wearing (64.6 ± 167.6 CFU) and non-denture-wearing normosalivation patients. Similarly, no significant difference in *C. albicans* colony counts was noted between the denture-wearing and non-denture-wearing hyposalivation patients.

## Discussion

The purpose of this study was to clarify the species of *Candida* that are most strongly associated with the development of *Candida*-associated atrophic glossitis because recent studies have suggested the importance of NAC pathogenesis in oral candidiasis. Our logistic regression analysis showed an association between increased amounts of *C. albicans* colonies and atrophic glossitis. However, no significant relationship between NAC and atrophic glossitis was observed.

With regard to the methods that are used to obtain samples from the oral cavity, although an oral rinse method is helpful for quantification, this method does not evaluate localized sites of infection [19]. In the present study, swab testing was used to evaluate local colonization on the dorsum of the tongue. Swab testing is simple to perform and is thought to be useful for quantitative estimation since the results obtained from rinsing and swab methods have been found to be correlated [20, 21]. A chromogenic media-based commercial system, CHROMagar™ Candida, was used for quantitative estimation; this selective and differential medium is used for the isolation and identification of *Candida* species [19]. The particular advantage of this system is its ability to detect mixed yeast infections in clinical samples [22]. Studies have suggested that *C. albicans*, *C. tropicalis*, *C. glabrata*, and *C. krusei* can be identified accurately using CHROMagar™ Candida [23–26]. Although it has been reported that the dark green appearance of *C. dubliniensis* can also be reliably distinguished from that of *C. albicans* [27], a more detailed method, such as molecular fingerprinting, is needed to discriminate between these two species since *C. albicans* and *C. dubliniensis* share many phenotypic characteristics [28]. In the present study, no molecular methods were applied; thus, *C. dubliniensis* colonies could not be distinguished from *C. albicans* on CHROMagar™ Candida plates. However, *C. dubliniensis* is an opportunistic oral pathogen that is typically isolated from patients infected with human immunodeficiency virus (HIV) [29]. Since the present study did not include any such patients, there is little concern about the potential for confusion.

*C. albicans*, but not NAC, proved to be associated with atrophic glossitis in the present study. This result was consistent with the observations reported by Terai et al. [5, 30], who found that *C. albicans* is predominantly detected as a single infection. The detection of NAC or a mixed flora consisting of *C. albicans* and NAC is therefore limited in patients with atrophic glossitis. Although *C. glabrata* was detected in 50 of the 135 (37.0%) patients with *Candida*, the second-most common isolated species after *C. albicans* (Table 2), no significant association was observed between *C. glabrata* and atrophic glossitis (Table 6). Since the clinical manifestation of erythematous candidiasis is related to the proteinase production capacity of *C. albicans* [31], a low level of protease secretion might explain why *C. glabrata* did not contribute to atrophic glossitis.

The virulence factors of *Candida* include adherence, evasion of the host defenses, and the invasion and destruction of host tissue [1]. Hyphae penetrate perpendicularly, traversing the surface epithelium up to the spinous cell layer [32]. The tip of the penetrating hyphae is known to be rich in proteinases, lipases, and several other enzymes. *C. glabrata* is unable to grow in filamentous forms and cannot invade the epithelial layer [33]. In contrast to other *Candida* species, the amounts of secreted aspartyl proteinases (SAPs) and phospholipases (PLs) produced by *C. glabrata* are extremely low [11, 34]. In *C. albicans* and *C. glabrata* mixed infections, *C. albicans* promotes the penetration of *C. glabrata* into the oral epithelium as *C. glabrata* cannot do so alone [33]. Once *C. albicans* penetrates the epithelial layer, the final step in the infection process is damage, which is characterized by the loss of the superficial epithelium [35]. *C. albicans* induces both apoptosis and necrosis in the oral epithelial cells [36]. In a study using reconstituted human oral epithelia, the hyphal elements of *C. albicans* invaded and led to marked disorganization of the epithelium [33]. In contrast, *C. glabrata* induced less epithelial damage [33]. Different levels of epithelial loss, namely, the severity of atrophic glossitis, may be related to the levels of disorganization of the epithelium. The removal of the keratin layer by oral dynamics, i.e., the movement of the tongue and other muscles, might be enhanced in certain situations, especially in patients with hyposalivation, due to the reduced lubricant function of the saliva. Erythematous candidiasis is the most prevalent variant in patients with hyposalivation [37]. Furthermore, in the present study, a logistic regression analysis revealed that a low SSFR was associated with atrophic glossitis (Table 6).

High vascularity is a characteristic finding of erythematous candidiasis in addition to epithelial atrophy or a lack of a keratinized epithelial surface layer [32]. One hypothesis regarding the mechanism underlying these findings is that erythematous candidiasis is accompanied by the activation of a partially reactive defense mechanism and that it may represent a clinical expression in response to candidal antigens [38, 39]. Because patients with systemic immunosuppressive diseases were excluded from the present study, the oral mucosa in the patients was presumed to be immunocompetent. Atrophic glossitis may be induced even in patients with normal immunity; as such, factors other than immunosuppression, such as hyposalivation, are necessary for the establishment of atrophic glossitis. With its thin epithelial layers, the oral mucous membrane might be sensitized to react to *Candida* and other virulence factors, thereby causing inflammation.

A chi-square test revealed that denture wearing was associated with the presence of atrophic glossitis (Table 4). The influence of salivation and denture wearing on *Candida* colony counts was evaluated using the Kruskal-Wallis H test. The number of *C. albicans* colonies (Fig. 2) was found to be significantly higher in the hyposalivation group than in the normosalivation group. This result is in accordance with the findings of a previous study, which demonstrated an inverse association between the salivary flow rate and *C. albicans* counts in saliva [40–42]. However, no significant difference was observed in the amounts of *C. albicans* colonies between the denture wearers and denture non-wearers. *C. glabrata* exhibits superior cell surface hydrophobicity and a greater tendency to adhere to the surface of denture acrylic resin than other *Candida* species [43]. In addition, NAC shows a greater ability to form biofilms on the surface of dental acrylic resin in comparison to *C. albicans*, while the combination of *C. albicans* and *C. glabrata* shows the highest biofilm-forming ability [44]. The morphological differences and growth patterns exhibited by *C. albicans* and *C. glabrata* may allow these species to occupy the same location within the oral cavity with limited competition for space, thereby allowing their co-existence [33]. Thus, the presence of *Candida* on the denture surface is a prominent etiological factor for denture stomatitis [45]. However, there seems to be no correlation between the colonization of *Candida* on the mucosal surface of the denture base and that on the dorsum of the tongue. Although denture wearers with hyposalivation are considered at substantial risk for oral candidiasis, increased *C. albicans* colony counts on the dorsum of the tongue is essential for *Candida*-related atrophic glossitis.

## Conclusion

*C. albicans* was associated with atrophic glossitis in xerostomia patients who had no systemic predisposing factors, indicating that *C. albicans* remains a treatment target for *Candida*-related atrophic glossitis.

## Abbreviations

NAC: Non-*albicans Candida*
RSFR: Resting whole salivary flow rate
SSFR: Stimulated whole salivary flow rate
